# Targeting differential energy substrate metabolism on a therapeutic ketogenic diet: a case report

**DOI:** 10.3389/fnut.2025.1623217

**Published:** 2025-08-20

**Authors:** Kasey J. Russell, Sarah E. O. Schwartz

**Affiliations:** ^1^Independent Researcher, Cambridge, MA, United States; ^2^Department of Psychology, Suffolk University, Boston, MA, United States

**Keywords:** ketogenic diet, glioblastoma, exercise, case study, metabolism

## Abstract

Ketogenic diets show promise in treating a range of serious illnesses, yet their clinical utility is often hindered by poor adherence and difficulty achieving targeted ketone levels. Recommendations for patients pursuing ketogenic diets provide detailed dietary guidance but largely neglect the potential role of exercise on ketone levels. In a case study of a glioblastoma patient following a ketogenic diet, we present data suggesting that intensity of exercise may predict ketone levels above and beyond energy expended through exercise, and we propose a possible mechanism for this observed association. It is our hope that this case study will highlight the need for further research investigating how intense exercise in particular may influence ketone levels for patients on therapeutic ketogenic diets, potentially enabling patients to achieve higher ketone levels or allowing ketosis on more liberal diets.

## 1 Introduction

More than 130 active clinical trials are investigating the impact of the ketogenic diet on a range of physical diseases as well as neurological and mental health disorders (clinicaltrials.gov). Although the ketogenic diet was originally developed to treat refractory epilepsy (1), recent research has identified the mechanisms through which it can reduce inflammation in the brain (2) and has indicated that it may have efficacy as an adjuvant treatment for a number of other serious illnesses including high-grade glioma (3–5). In addition, it has been shown to heighten adaptive immunity (6), suggesting promise as an adjuvant to immunotherapies in general.

While there is individual variability, higher concentrations of ketone bodies are generally believed to be more therapeutic, with some studies showing a significant increase in seizure control for blood ketone concentrations above 4 mM (7). In the context of cancer treatment, ketogenic diets may serve as an adjuvant treatment to chemoradiation to slow tumor growth both by limiting glucose availability and through inherent anti-cancer properties of ketones (3), and a target ratio of glucose to ketone concentration (glucose ketone index, GKI) of less than 1 has been proposed (8). For treatment of high-grade glioma, one study showed that higher ketone levels were associated with greater odds of survival (9), and *in vitro* investigations have shown antitumor effects from physiologically relevant concentrations of ketones, even in the presence of high glucose (3). Adherence to the diet poses a key challenge, however, not only to reaching high ketone levels, but even to achieving and maintaining ketosis (10). Many clinical studies have observed that adults in particular have difficulty achieving high ketone levels (10), challenging efforts to attain or even identify optimum blood ketone levels for the treatment of many diseases.

Metabolic studies have demonstrated that the relative utilization rate of different energy substrates (e.g. muscle glycogen, muscle triglycerides, plasma free fatty acids) depends on exercise intensity (11). This differential utilization of energy substrates can be modified by long-term adaptation to a ketogenic diet (12). Whereas researchers have previously investigated how this keto-adaptation may enhance exercise performance (12), in our case we are interested in the inverse: investigating how keto-adapted patients may be able to tailor their exercise regimen to help increase ketone levels, with the ultimate goal being prolonged survival (in the case of glioma) or another therapeutic metric.

In this case study of a glioblastoma patient following a ketogenic diet, we present data suggesting that intense exercise in particular may help increase daily ketone levels in keto-adapted individuals, with the goal of motivating further research in this area. All data were collected and analyzed by the patient, author K.J.R., and his spouse, author S.S., and are presented here without de-identification in the hope that they may benefit others facing similar challenges. Since the patient was simply following treatment guidelines and is willingly publishing his own data for this case study, ethics board approval was not sought. The patient, a 44-year-old, White, European-American man, 184 cm tall with body mass of 68 kg, presented with a seizure that was determined to be caused by a glioma. Surgical resection and subsequent pathology confirmed a diagnosis of glioblastoma, isocitrate dehydrogenase (IDH)-wildtype, with unmethylated MGMT promoter sequence. The patient took antiepileptic medications (levetiracetam and lacosamide) but did not take steroids at any point in the data collection period, and his body mass was relatively stable (+/– 2 kg).

In consultation with a cancer center dietician, the patient initiated a ketogenic diet in the days leading up to the start of standard chemoradiation treatment (13). While the traditional therapeutic diet for refractory epilepsy has a fat to protein plus net carbohydrate ratio of 4:1 (1), the dietician recommended consuming 60–70 g/day protein and a total of 2,200–2,500 kcal/day with maximum 20 g/day net carbohydrates to maintain sufficient protein intake for tissue repair in the context of chemoradiation, resulting in a dietary fat ratio of 2.25:1 to 3:1. See Methods for a more detailed description of diet.

**Figure 1:**
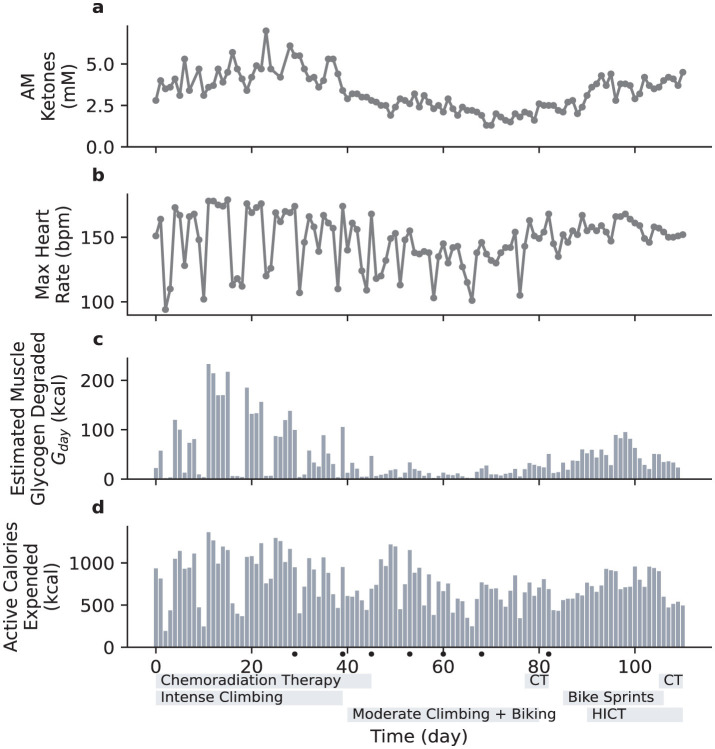
Daily variation of blood ketone levels compared with various metrics associated with physical exercise. The duration of radiation therapy is indicated with a gray bar below the horizontal axis. Days with seizures are indicated with dots below the horizontal axis. CT, chemotherapy. HICT, high-intensity circuit training. **(a)**, Daily blood ketone levels (first measurement each morning). **(b)**, Daily maximum heart rate. **(c)**, Estimated muscle glycogen degraded, *G*_*day*_. **(d)**, Active caloric expenditure, as measured by Apple Watch.

Given the challenges adults tend to have in achieving high ketone levels (10), it was somewhat surprising that the patient rapidly achieved ketone levels at or above 4 mM (Figure 1) and GKI at or below 1 (Supplementary material Extended Data Figure 1). The patient was exercising regularly (indoor rock climbing, consisting of multiple 2–3 min intense climbs with breaks in between over the course of 45–60 min), and examination of the data indicated an association between ketone levels and exercise with a 1 day lag period: exercising Monday through Friday was associated with higher ketone levels Tuesday through Saturday, while ketone levels were lower on Sundays and Mondays. After a series of seizures appeared to be triggered by intense climbing in the context of reduced seizure threshold due to chemoradiation treatment, the intensity of climbing was reduced, and the patient observed a subsequent reduction in ketone levels (Figure 1). Replacement exercises such as walking and stationary bicycle riding (45–60 min sessions of moderate cycling) were initiated, but ketone levels failed to rebound, even when similar levels of calorie deficit were achieved (Supplementary material Extended Data Figure 2). Time-restricted eating (14) also failed to have a significant impact.

Since the primary change in exercise routine was a marked reduction in intensity, we hypothesized that intensity of exercise was an important factor governing ketone levels. To test this, the patient attempted to develop a different high intensity exercise regimen that would be less likely to result in seizures. On Day 85 the patient modified stationary cycling to include brief high intensity components, specifically cycling for 30 min with 1-min sprints starting at minutes 5, 10, 15, 20, and 25 (in contrast to the previous 45–60 min at a continuous moderate pace), and ketone levels increased (Figure 1). On Day 90, the patient added 7-min high-intensity circuit training sessions [HICT, Klika and Jordan (15), a.k.a. “New York Times 7-min Workout"] twice per day in the morning and evening, resulting in three brief but intense bouts of exercise spaced throughout the day. Ketone levels returned to around 4 mM. Due to scheduling constraints, the stationary bicycle session was replaced with a third HICT session on Day 106, and ketone levels remained high.

These results provided preliminary support for an association between intensity of the exercise and ketone levels in the context of keto-adaptation. To quantitatively examine the relative contributions of both exercise-related and dietary predictors of ketone levels, we then conducted a multivariate regression analysis including: previous day's ketone levels, dietary fat ratio (see Supplementary material Extended Data Figure 2), calories ingested, calories expended, and maximum heart rate (as a rough proxy for exercise intensity). The first morning ketone measurement of the day was used to reduce the short-term influences of food and exercise on ketone levels. This measurement (AM Ketone) was typically not the highest of the day; see Supplementary material Extended Data Figures 3, 4. The overall model was statistically significant [*R*^2^ = 0.70, *F*_(5, 105)_ = 46.63, *p* < 0.001]. Given that it typically takes a person several days to enter ketosis, it was not surprising that the strongest predictor was the previous day's ketone levels (β = 0.68, *p* < 0.001). Maximum heart rate also emerged as a significant predictor (β = 0.23, *p* = 0.009). Dietary fat ratio and calories ingested were not significant, likely due to the tightly controlled nature of the diet, and active calories expended also failed to reach significance (see Supplementary material Extended Data Table 1 for full results).

The fact that maximum heart rate emerged as a significant predictor, above and beyond active calories expended, suggest that exercise intensity may play a role in predicting ketone levels. However, a single measurement of maximum heart rate cannot fully describe a single workout, let alone an entire day of exercise. While there remains much unknown about the various mechanisms at play, muscle glycogen use is one potential mechanism that has been a target of previous studies. These studies have shown that muscle glycogen use depends exponentially on exercise intensity (16), and there is an established link between liver glycogen and post-exercise ketone levels (17). We therefore drew on existing experimental literature (11, 18, 19) while attempting to account for adaptation to a ketogenic diet (12, 20) to develop a rough model of muscle glycogen degradation based on a time-integrated exponential function of measured heart rate (see Methods for details).

We then substituted this estimate of daily muscle glycogen degradation for maximum heart rate in our multiple regression model. The overall model was statistically significant, accounting for a greater percentage of the variance than the model with maximum daily heart rate [*R*^2^ = 0.77, *F*_(5, 105)_ = 46.63, *p* < 0.001]. Again, the strongest predictor was the previous day's ketone levels (β = 0.64, *p* < 0.001). Estimated glycogen degradation was also a significant predictor (β = 0.32, *p* < 0.001). Active calories expended, dietary fat ratio and calories ingested all failed to reach significance (see Methods for detailed model and Supplementary material Extended Data Table 2 for full results).

Although the exercise intensity dependence we observed has not previously been reported, it may be consistent with existing literature when viewed in aggregate. Muscle glycogen replenishment is a high metabolic priority (17, 21), meaning that it may compete with other uses for exogenous carbohydrates and protein, even for individuals on a ketogenic diet. Additionally, there is a well-known post-exercise increase in blood ketones [i.e., the Courtice-Douglas effect (22)] that appears to depend on the degree of liver glycogen depletion (17, 23). Previous research has found no strong dependence of post-exercise ketosis on exercise intensity (22, 23), but in those studies the participants were following a conventional (not ketogenic) diet. When on a ketogenic diet for several weeks, muscles adapt to conserve glycogen (12, 20) [also known as glycogen sparing (24)], greatly reducing glycogen usage at low and medium exercise intensities. However, because glycogen use increases exponentially with exercise intensity, we would still expect it to be significant during high intensity exercise (11, 16). It is therefore possible that we are observing an intensity-dependent version of the Courtice-Douglas effect that is specific to the context of keto-adaptation. This speculative assessment highlights the need for more research investigating exercise intensity and potential mechanisms in the context of individuals on a long-term ketogenic diet.

Clinical research on the therapeutic effects of the ketogenic diet have long been hindered by the challenges adult participants experience in achieving elevated ketone levels. A recent paper concluded that although high ketone levels can be achieved in pediatric populations, “high levels of nutritional ketosis are unobtainable in adults” (10). While there clearly are innate differences in metabolism between children and adults, perhaps differences in behavior also play a role (e.g., kids are more likely to start sprinting across a playground).

Speaking as a patient pursuing a ketogenic diet for therapeutic purposes, there are abundant resources with different suggestions for macronutrient guidelines, fasting, and exogenous ketone supplementation [e.g., Ref. (1), charliefoundation.org]. However, few discuss the role of exercise, and those that do simply recommend exercise broadly without attention to type or intensity of exercise. While intense exercise understandably is not possible for all patients, for some it could offer a way to achieve desired ketone levels on a less restrictive diet or at least mitigate the impact of “cheat days” on ketone levels. It should be noted, however, that in the current study the patient was on a rigorous ketogenic diet, and our data do not provide evidence that exercise alone can increase ketosis. Importantly, exercise demonstrates health benefits irrespective of its impact on ketone levels, including as a potential adjuvant treatment for glioma (25). Exercise has also been shown to be an effective treatment for depression (a common comorbidity for patients with glioma and other serious diseases), with impacts proportional to the exercise intensity prescribed (26). The benefits of intense exercise, however, must be weighed against the potential for adverse events (e.g., seizures) when engaging in intense exercise, especially in the context of chemoradiation.

More generally, our results indicate the need for additional research on how exercise intensity may influence ketone levels specifically in keto-adapted individuals. There is substantial variability in metabolism among individuals, and there were a number of factors beyond exercise and diet that likely also influenced ketone levels in this patient, radiation and chemotherapy treatments in particular. While the Apple Watch used for activity tracking allowed for a non-invasive, near-continuous monitoring of both heart rate and active calorie expenditure, its accuracy at measuring caloric expenditure is known to be questionable (27). Further research is therefore needed to draw causal conclusions, identify mechanisms of action, and ideally develop specific guidelines for exercise regimens that can elevate ketone levels. It is our hope that this work can enable easier adherence to this promising dietary intervention, opening up new pathways for research and clinical practice.

## 2 Methods

### 2.1 Data collection

All ingested food was logged using an online application (https://cronometer.com) and in most instances was massed to the nearest gram. Logged values were used to estimate dietary macronutient intake as well as total caloric intake. An Apple Watch (Series 9, Apple Inc., California, United States) was used to track a variety of physiologic parameters including heart rate and active caloric expenditure (i.e. calories expended from activity beyond basal metabolic expenditure). Blood ketone and glucose were monitored with a commercially-available meter (GK+, Keto-Mojo, California, United States). See Data Availability for access to all data (including full dietary log).

### 2.2 Diet

The patient's diet was relatively consistent over the data collection period. On a typical day, he ingested about 12 g/day of MCT oil, 50 g/day of butter, and 80 g/day of olive oil, supplemented with heavy cream, mayonnaise, avocado, coconut milk, and small amounts of coconut butter. Most protein was from meat, fish, eggs, and cheese, with a small amount from nuts. The main vegetables consumed were kale and spinach, with a small amount of tomato and garlic. Fruit consumption was minimal, mainly consisting of blackberries or raspberries used as garnish. Flaxseed and chia seeds provided supplemental dietary fiber, with occasional consumption of magnesium citrate (dissolved in water) and potassium chloride (included in lite salt) for additional electrolytes. Vitamin D was the only daily dietary vitamin taken.

### 2.3 Data analysis

Quantitative analyses were performed in Python using the statsmodels package. Ordinary least square regressions calculated *K*_*i*_, the AM ketone levels for day *i*. The equation for the preliminary analysis using maximum heart rate is given by:


(1) $$\begin{array}{l} K_{i}=b_{C}+b_{K}K_{i-1}+b_{H}H_{i-1}+b_{E}E_{i-1}+b_{A}A_{i-1}+b_{F}F_{i-1}+\varepsilon_{i}, \end{array}$$

where subscripts *i* and *i*−1 denote a given day and the previous day, respectively; *b*_*C*_ is the fitted constant/intercept; *b*_*K*_, *b*_*H*_, *b*_*E*_, *b*_*A*_, and *b*_*F*_ are coefficients for the various predictors; *K* is the AM ketone level; *H* is the maximum heart rate; *E* is the food energy ingested; *A* is the active calories expended (i.e., “active calories” as measured by the Apple Watch); *F* is the dietary fat ratio; and ε_*i*_ is the residual/error for the given day. See Supplementary material Extended Data Table 1 for full results of this regression.

The equation for the regression using estimated glycogen degradation is given by:


(2) $$\begin{array}{l} K_{i}=b_{C}+b_{K}K_{i-1}+b_{G}G_{i-1}+b_{E}E_{i-1}+b_{A}A_{i-1}+b_{F}F_{i-1}+\varepsilon_{i}, \end{array}$$

where all terms are the same as from Equation 1 with the exception of *b*_*G*_ and *G*_*i*−1_, which replaced *b*_*H*_ and *H*_*i*−1_, respectively. *G* is the estimated glycogen degraded (defined below in Equation 3). Full results from this regression are shown in Supplementary material Extended Data Table 2, and summary plots are shown in Supplementary material Extended Data Figure 5.

We estimated *G*_*i*_, the total muscle glycogen degradation for day *i*, using time-varying heart rate data from the Apple Watch, *h*(*t*). Estimating anaerobic energy substrate usage from an aerobic measure of exertion is approximate, especially in the case of a subject adhering to a ketogenic diet for an extended period of time (12, 20). However, it allows for a cheap, non-invasive, and scalable way to provide actionable feedback to patients, so there is merit to this approach despite the error involved.

The value of *G*_*i*_ is estimated by the following time integral over day *i*:


(3) $$\begin{array}{l} G_{i}=M_{B}\underset{i}{\int }g\left(t\right)dt, \end{array}$$

where *M*_*B*_ = 68 kg is the subject's body mass and *g*(*t*) is the heart-rate-dependent muscle glycogen degradation rate. Muscle glycogen usage has been shown to increase exponentially with exercise intensity (16), so we approximated *g*(*t*) as an exponential function of heart rate, *h*(*t*) (see Supplementary material Extended Data Figures 6, 7):


(4) $$\begin{array}{l} g\left(t\right)=be^{ah\left(t\right)/h_{M}}. \end{array}$$

Here, *h*_*M*_ = 180 bpm is the subject's estimated maximum heart rate (no VO_2_max test was performed because of seizure risk, but the highest measured heart rate was 179 bpm on Day 15). Constants *a* = 16.8 and *b* = 3.5 × 10^−8^ kcal/min are derived from fitting an exponential function to muscle glycogen degradation rate data from Romijn et al. (18) after attempting to adjust for keto-adaptation of muscle (20). The muscle glycogen degradation rates for subjects not on a carbohydrate-restricted diet at 65 and 85% of VO_2_max were extracted from figure 8 of Romijn et al. (18) and found to be 80 and 175 cal/kg/min, respectively. Phinney et al. (20) found a four-fold reduction in muscle glycogen degradation at 65% VO_2_max for keto-adapted subjects, allowing uncompromised athletic performance despite the significant carbohydrate restriction of their diets. Although no similar data was available at higher VO_2_max, athletic performance was compromised at high intensity indicating that even in the context of keto-adaptation, glycogen is still the dominant energy substrate for high intensity exercise. We therefore used a value of 80/4 = 20 cal/kg/min for 65% VO_2_max and left the value for 85% of VO_2_max from Romijn et al. (18) (175 cal/kg/min) unchanged. The conversion between %VO_2_max and % maximum heart rate was performed using the linear mapping from Swain et al. (19), i.e., 65 and 85% VO_2_max correspond to 79 and 91% of maximum heart rate, respectively.

## Data Availability

The datasets presented in this study can be found in online repositories. The names of the repository/repositories and accession number(s) can be found at: https://doi.org/10.17632/x6cxkxf3w2.1.
