# Supplementary material for: Targeting differential energy substrate metabolism on a therapeutic ketogenic diet: a case report
Source: Front Nutr. 2025 Aug 20;12:1623217. doi: 10.3389/fnut.2025.1623217 (PMC12404966; doi:10.3389/fnut.2025.1623217)
Supplement: Supplementary file 1 [file Supplementary_file_1.pdf]

Appendix: Targeting differential energy substrate metabolism on a therapeutic ketogenic diet: A case report

Kasey J. Russell[1*] and Sarah E. O. Schwartz[2]

[1*]Currently on medical leave. Cambridge, 02140, MA, USA.
[2]Department of Psychology, Suffolk University, Boston, 02108, MA, USA.

*Corresponding author(s). E-mail(s): kasey.russell@gmail.com;
Contributing authors: seoschwartz@suffolk.edu;

Extended Data

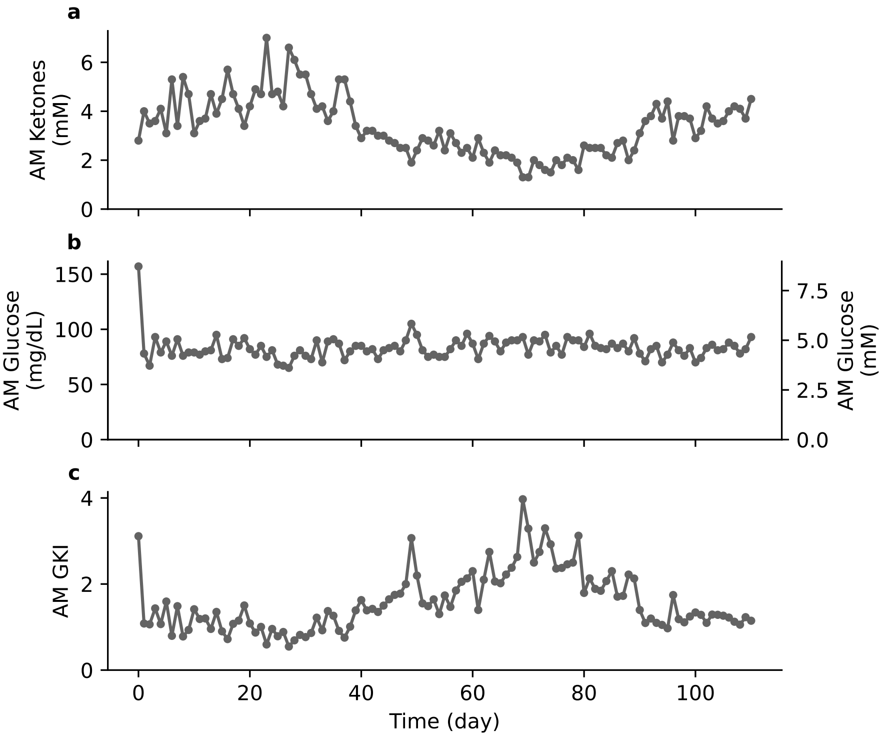

Extended Data Fig. 1 Daily variation of first morning measurements of blood ketone levels, blood glucose, and computed glucose/ketone index, GKI. **a**, Blood ketone levels. **b**, Blood glucose levels. **c**, GKI.

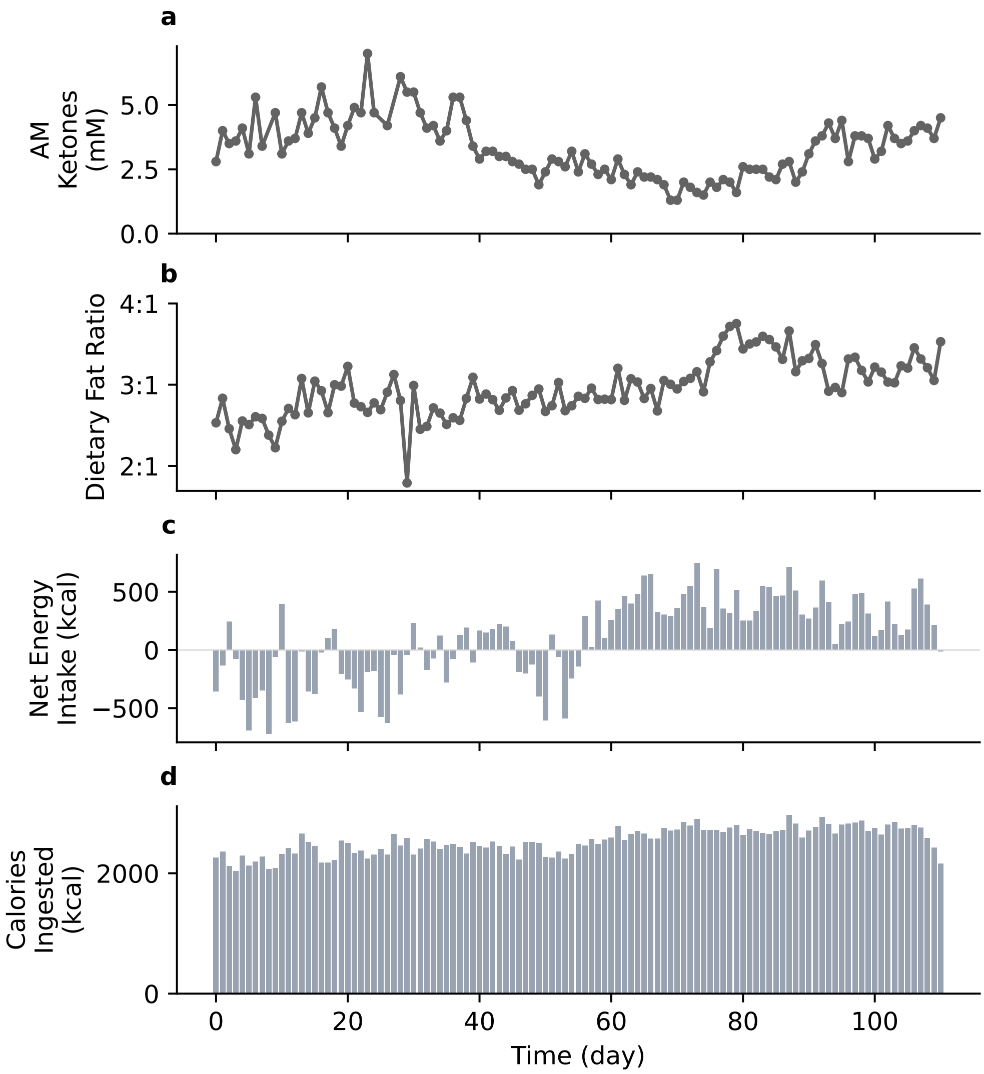

Extended Data Fig. 2 **a**, AM Ketone levels. **b**, Dietary fat ratio: mass of fat to the sum of masses of protein and net carbohydrate. **c**, Daily net caloric intake (calories ingested minus the sum of active calories expended and basal metabolic rate, assumed to be 1676 kcal/day). **d**, Daily caloric intake.

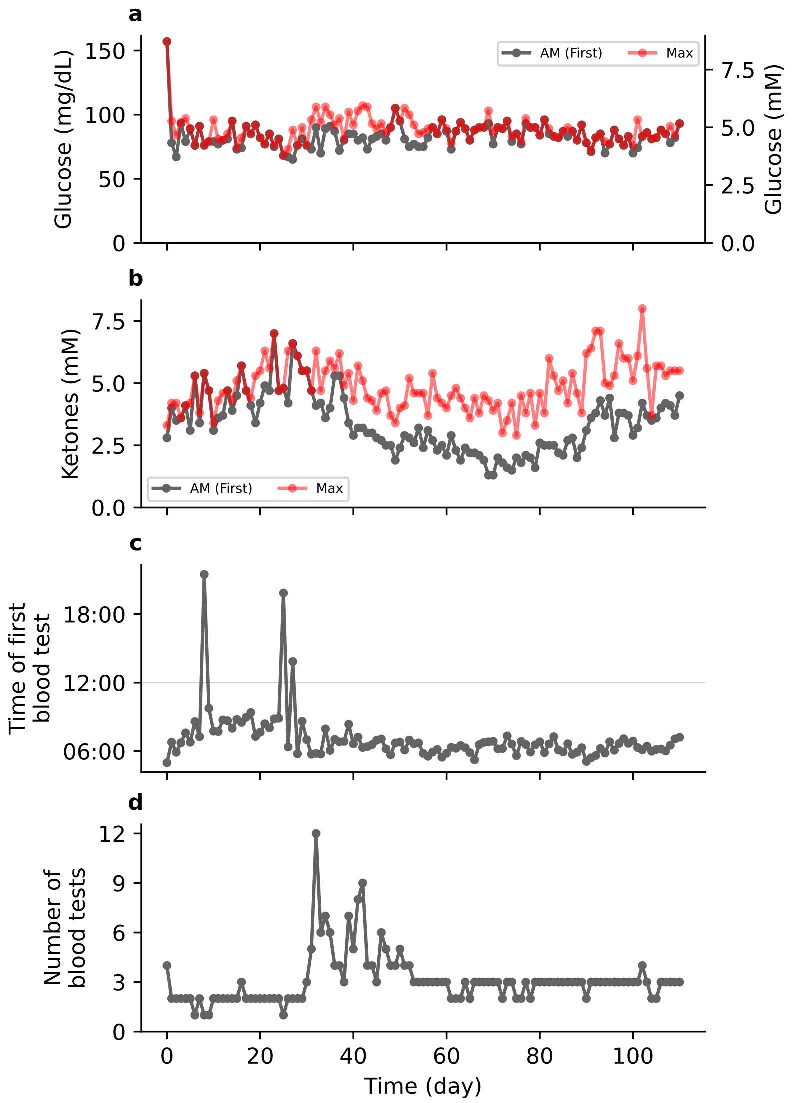

Extended Data Fig. 3 Various blood test characteristics. **a**, Glucose: comparison between first measurement of each day and the daily maximum. **b** Ketones: comparison between first measurement of each day and the daily maximum. **c**, Time of day of first blood test. (The three days with first tests after 12:00 were excluded from all analyses.) **d**, Number of blood tests per day.

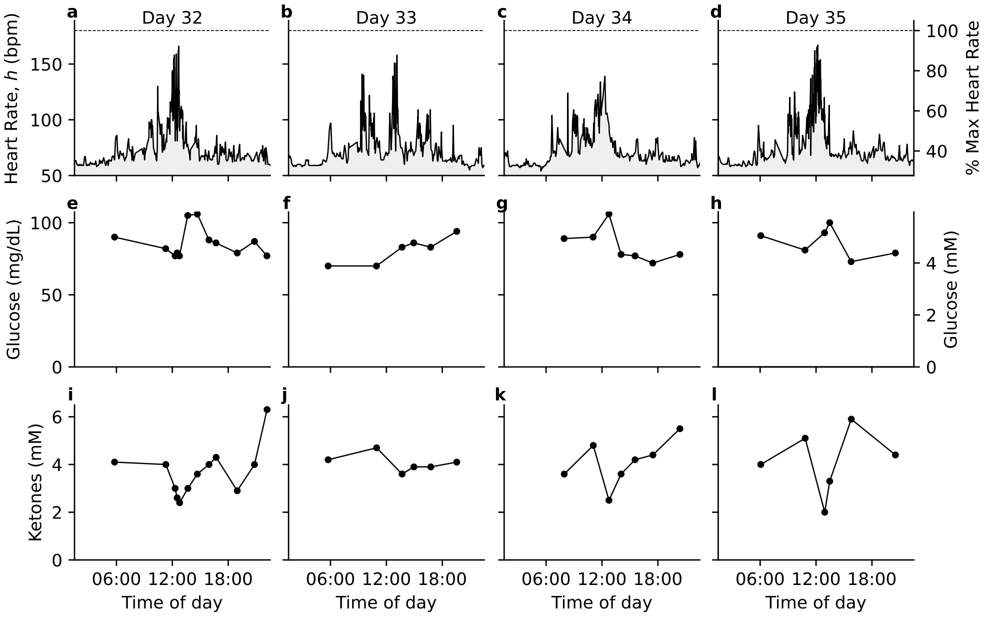

Extended Data Fig. 4 Continuous heart rate versus time and all blood tests measured across four consecutive days (Days 32-35). **a-d**, Heart rate. **e-h**, Glucose. **i-l**, Ketones.

Extended Data Table 1 Regression table from preliminary analysis predicting AM ketone level with maximum heart rate

	β	B	SE	95% CI		p
				LL	UL	
Intercept	0.00	0.86	0.87	-0.88	2.59	.328
AM Ketones Prev. Day (mM)	0.68	0.66	0.06	0.54	0.79	<.001
Max Heart Rate Prev. Day (kbpm)	0.23	12.30	4.61	3.15	21.46	.009
Calories Ingested Prev. Day (Mcal)	-0.14	-0.70	0.42	-1.53	0.13	.097
Active Calories Prev. Day (Mcal)	0.08	0.34	0.36	-0.36	1.05	.337
Fat Ratio Prev. Day	-0.01	-0.02	0.25	-0.51	0.48	.949
$R^2 = .70$						

β, standardized coefficient. B, unstandardized coefficient. *SE*, standard error. *CI*, confidence interval. *LL*, lower limit. *UL*, upper limit.

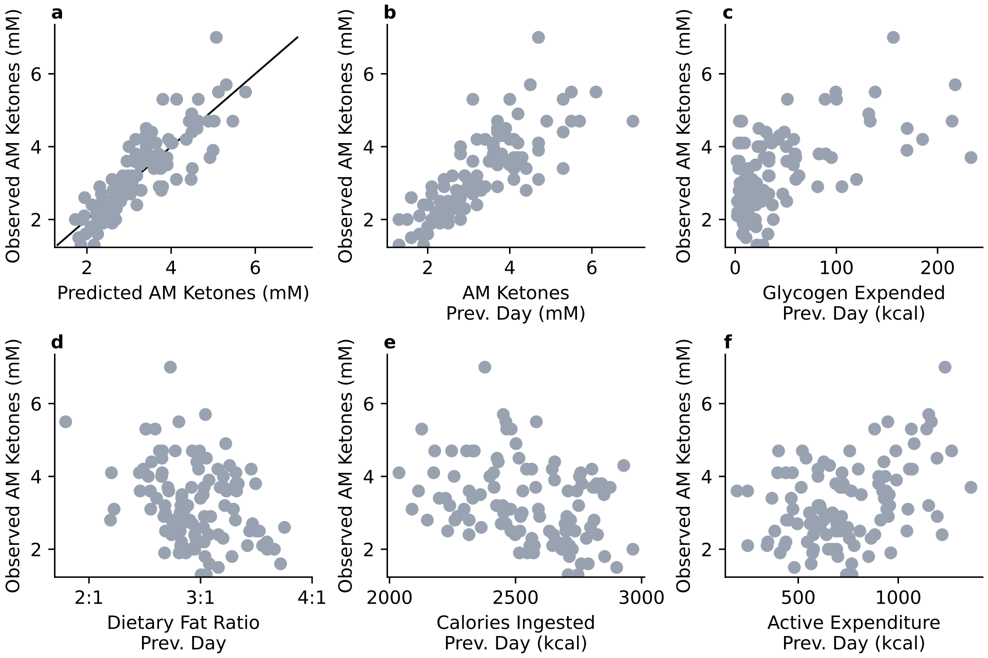

Extended Data Fig. 5 Summary plots of the regression model using estimated glycogen degradation. (See Extended Data Table 2 for full results.) **a**, Overall performance of the model showing observed versus predicted AM ketone levels. **b**, Observed ketone level versus ketone level from the previous day. **c**, Observed ketone level versus the previous day's estimated glycogen using Eq. **??**. **d**, Observed ketone level versus dietary fat ratio from the previous day. **e**, Observed ketone level versus calories ingested the previous day. **f**, Observed ketone level versus active calories expended the previous day.

Extended Data Table 2 Regression table predicting AM ketone level with estimated glycogen degradation

	β	B	SE	95% CI		p
				LL	UL	
Intercept	0.00	2.13	0.88	0.39	3.87	.017
AM Ketones Prev. Day (mM)	0.64	0.62	0.06	0.50	0.74	<.001
Glycogen Use Prev. Day (Mcal)	0.32	6.92	1.62	3.71	10.14	<.001
Calories Ingested Prev. Day (kcal)	-0.08	0.00	0.00	0.00	0.00	.270
Active Calories Prev. Day (kcal)	0.03	0.00	0.00	0.00	0.00	.714
Fat Ratio Prev. Day	-0.02	-0.07	0.24	-0.54	0.40	.763
R^2 = .73						

β, standardized coefficient. B, unstandardized coefficient. SE, standard error. CI, confidence interval. LL, lower limit. UL, upper limit.

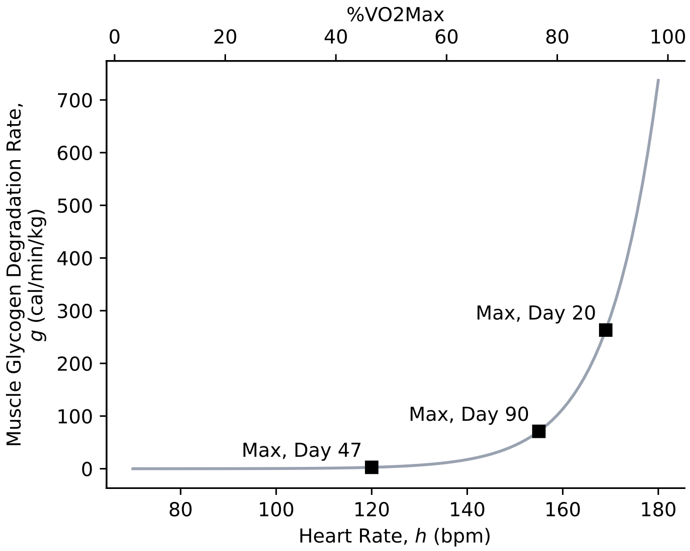

Extended Data Fig. 6 Plot of Eq. **??**: a rough model of muscle glycogen degradation rate, g, versus subject's heart rate, h, and estimated %VO2 Max. Maximum g and h from each of the days in Extended Data Fig. 7 are labeled.

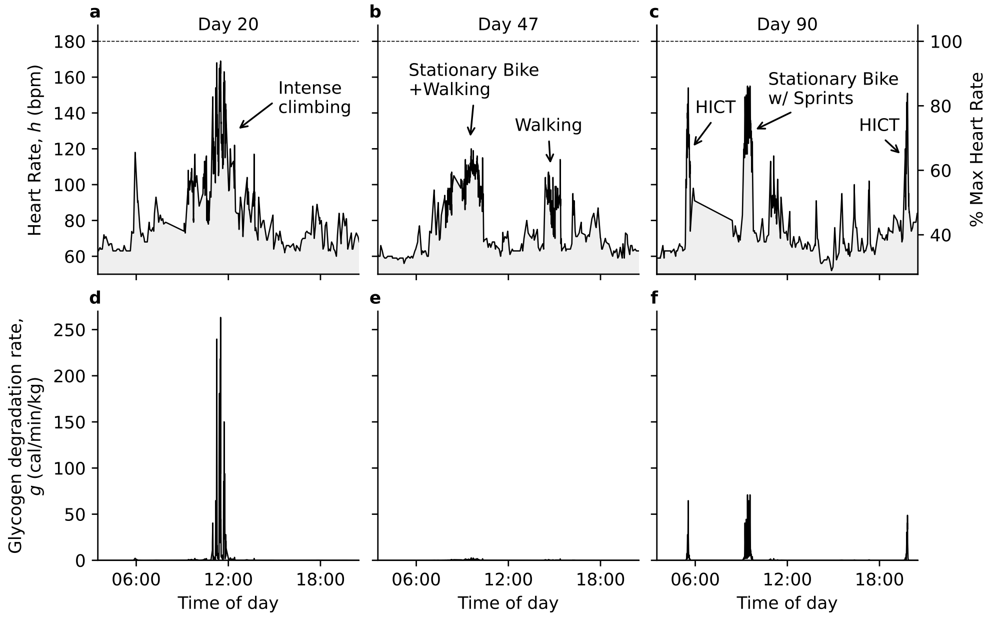

Extended Data Fig. 7 Heart rates and estimated muscle glycogen degradation rates for three representative days: Days 20, 47, and 90. **a**, **b**, **c**: Measured heart rate, h, for the three days, respectively. The horizontal line at 180 bpm indicates maximum heart rate, h_M. **d**, **e**, **f**: Estimated muscle degradation rate, g, calculated using Eq. **??** for the three days, respectively. See Extended Data Fig. 6 for plot of the estimated exponential dependence of g on h.
